# Epistasis-Driven Evolution of the SARS-CoV-2 Secondary Structure

**DOI:** 10.1007/s00239-022-10073-1

**Published:** 2022-09-30

**Authors:** Mahsa Alemrajabi, Ksenia Macias Calix, Raquel Assis

**Affiliations:** 1 Department of Physics, Florida Atlantic University, Boca Raton, FL 33431 USA; 2 Department of Electrical Engineering and Computer Science, Florida Atlantic University, Boca Raton, FL 33431 USA; 3 Institute for Human Health and Disease Intervention, Florida Atlantic University, Boca Raton, FL 33431 USA

**Keywords:** Epistasis, Compensatory evolution, SARS-CoV-2, Coronavirus, Secondary structure

## Abstract

Epistasis is an evolutionary phenomenon whereby the fitness effect of a mutation depends on the genetic background in which it arises. A key source of epistasis in an RNA molecule is its secondary structure, which contains functionally important topological motifs held together by hydrogen bonds between Watson–Crick (WC) base pairs. Here we study epistasis in the secondary structure of severe acute respiratory syndrome coronavirus 2 (SARS-CoV-2) by examining properties of derived alleles arising from substitution mutations at ancestral WC base-paired and unpaired (UP) sites in 15 conserved topological motifs across the genome. We uncover fewer derived alleles and lower derived allele frequencies at WC than at UP sites, supporting the hypothesis that modifications to the secondary structure are often deleterious. At WC sites, we also find lower derived allele frequencies for mutations that abolish base pairing than for those that yield G·U “wobbles,” illustrating that weak base pairing can partially preserve the integrity of the secondary structure. Last, we show that WC sites under the strongest epistatic constraint reside in a three-stemmed pseudoknot motif that plays an essential role in programmed ribosomal frameshifting, whereas those under the weakest epistatic constraint are located in 3’ UTR motifs that regulate viral replication and pathogenicity. Our findings demonstrate the importance of epistasis in the evolution of the SARS-CoV-2 secondary structure, as well as highlight putative structural and functional targets of different forms of natural selection.

**Supplementary Information:**

The online version contains supplementary material available at 10.1007/s00239-022-10073-1.

## Introduction

**Fig. 1:**
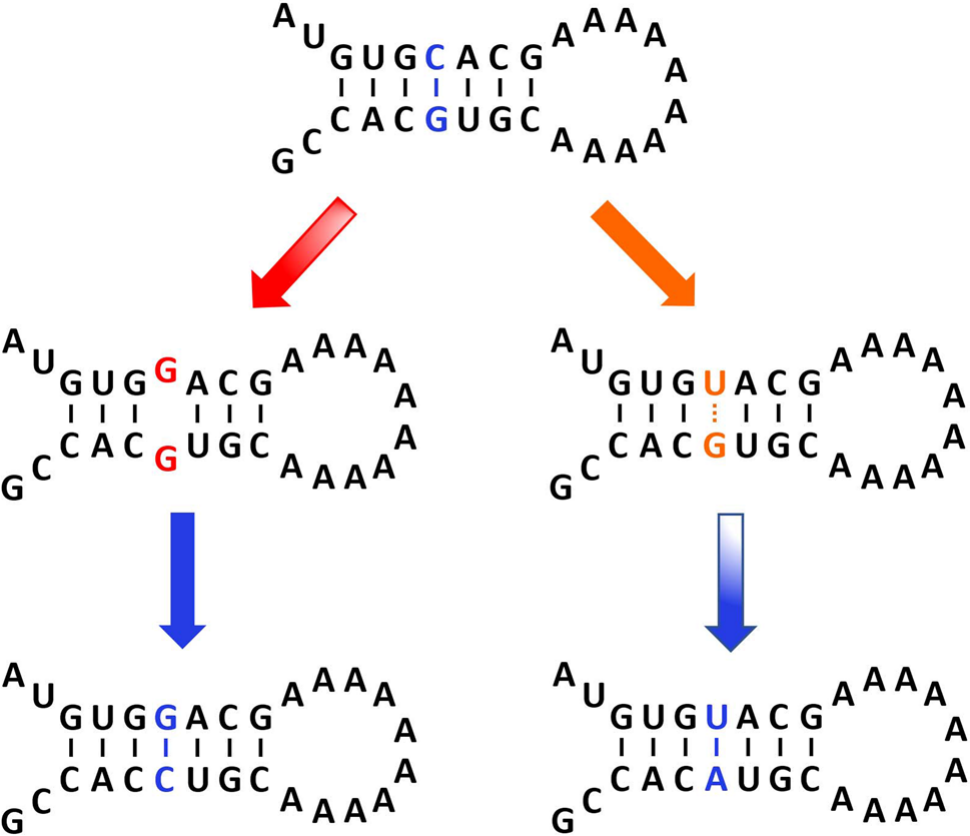
Compensatory evolution in a stem loop of a secondary structure. The ancestral stem loop (top) contains a WC base pair (blue). A mutation of one nucleotide (G) results in either UP nucleotides (middle left) or a weak G·U wobble base pair (middle right). In both scenarios, a compensatory mutation of the opposing nucleotide restores WC base pairing (bottom), though it may have more time to arise through the more stable G·U intermediate (bottom right) (Color figure online)

**Fig. 2:**
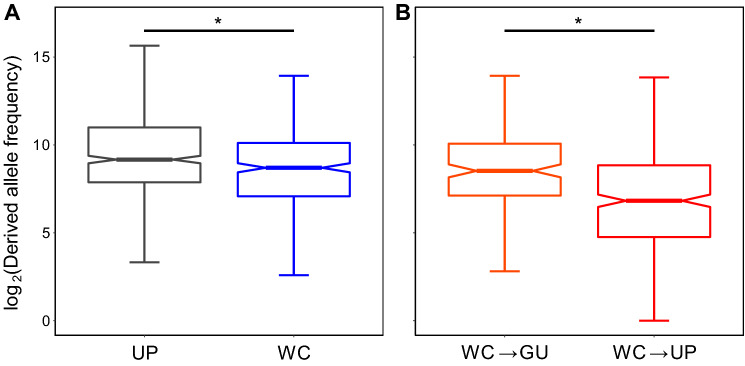
Derived allele frequencies at WC and UP sites. Distributions of derived allele frequencies for *A*) all ancestral WC and UP sites and *B*) WC → GU and WC → UP mutations. *$P<10^{-4}$ (see “Methods” section for details)

Mutations introduce new genetic variants, or alleles, into a population. Two opposing forms of natural selection may influence the evolutionary fates of these alleles—negative selection, a pervasive force that decreases the frequencies of deleterious alleles in a population and leads to evolutionary conservation (Eyre-Walker and Keightley 2007), and positive selection, a rarer force that increases the frequencies of beneficial alleles in a population and leads to evolutionary divergence and adaptation (Orr 2005; Eyre-Walker and Keightley 2007). Epistasis is an evolutionary phenomenon whereby the fitness effect of an allele at a locus, and therefore the type of selection that acts on it, depends on its interactions with alleles at other loci (Phillips 2008). There are many potential causes of epistasis, including interactions among genes and proteins that participate in the same biological pathways or functional modules (Lehner 2011). For example, mutations that alter the tertiary structures of proteins can hinder or enhance their binding affinities to other proteins, ligands, and molecules (Bloom et al. 2007; Starr and Thornton 2016). A simple and common source of epistasis in an RNA molecule is its secondary structure (Rousset et al. 1991; Kirby 1995), in which Watson–Crick (WC) base pairs A·U and G·C compose well-defined topological motifs that facilitate critical functions in the cellular environment (Mortimer et al. 2014). Consequently, mutations arising at WC base-paired sites are often deleterious, with those that abolish base pairing generally more disfavored than those that produce weaker G·U wobble base pairs (Rousset et al. 1991; Olsthoorn et al. 1994; Kirby 1995; Stephan 1996; Innan and Stephan 2001; Dutheil et al. 2010; Meer et al. 2010; Assis 2014). In such a scenario, WC base pairing may be restored via either a “back” mutation to the original allele at the same site or a “compensatory” mutation to a new allele at the opposing site (Fig. 1) (Rousset et al. 1991; Kirby 1995), each of which may fully recover the thermodynamic stability and functionality of the secondary structure (Olsthoorn et al. 1994; Kirby 1995; Berkhout et al. 1997; Chen and Stephan 2003; Dutheil et al. 2010; Meer et al. 2010).

Epistasis between WC base pairs of secondary structures likely plays a major role in the evolution of RNA viruses. For one, secondary structures are critical to all stages of viral life cycles and functions, such as evasion of host immune responses (Harrison and Lever 1992; Fernández et al. 2013; Ruelas and Greene 2013; Witteveldt et al. 2014; Napthine et al. 2017; Smyth et al. 2018). In coronaviruses, several well-described topological motifs guide binding of viral and host proteins during replication and translation (Brian and Baric 2005; Liu et al. 2009; Yang and Leibowitz 2015; Wacker et al. 2020). Therefore, it is not surprising that secondary structures of many RNA viruses, including coronaviruses, are highly conserved (Berkhout 1992; Harrison and Lever 1992; Berkhout et al. 1997; Liu et al. 2009; Fernández et al. 2013; Assis 2014; Rangan et al. 2020; Wacker et al. 2020). For example, there is extensive conservation of topological motifs located in the 5$^{\prime }$ regions of mouse MHV-A59, bovine BCoV, human MERS-CoV, human SARS-CoV, and human SARS-CoV-2 coronaviruses (Chen and Olsthoorn 2010; Guan et al. 2012; Yang and Leibowitz 2015; Wacker et al. 2020). However, what is surprising is that this conservation occurs against a backdrop of viral mutation and evolutionary rates that are orders of magnitude higher than those of any known life form (Drake 1993; Lynch 2010; Duffy 2018). Thus, epistasis between WC base pairs of secondary structures can result in simultaneously strong conservation of secondary structures and weak conservation of their underlying nucleotides. A striking example involves the R regions of human immunodeficiency virus type 2 (HIV-2) and mandrill simian immunodeficiency virus (SIV), which have nearly identical secondary structures despite their only 40% sequence conservation (Berkhout 1992). Further, a recent analysis in SARS-CoV-2 uncovered evidence of positive selection on alleles that perturbed the secondary structure without impacting the tertiary structures of any overlapping proteins (Berrio et al. 2020). Thus, adaptation of an RNA virus may proceed through modifications of its secondary structure, perhaps introducing new topological motifs for tackling emerging challenges to its evolutionary success.

Given the importance of secondary structures in RNA viruses, probing their evolution is likely to enhance understanding of functionally relevant sites. It is therefore not surprising that there has been a recent explosion of interest in interrogating the evolution of the SARS-CoV-2 secondary structure (Berrio et al. 2020; Rangan et al. 2020; Wacker et al. 2020). Even before publication of the first experimentally determined secondary structure of SARS-CoV-2 in late 2020 (Wacker et al. 2020), researchers used secondary structure prediction algorithms to assay its evolutionary conservation with other coronaviruses (Rangan et al. 2020; Simmonds 2020). The first of these studies showed that predicted secondary structures tend to be enriched for short sequences with perfect conservation across coronaviruses (Rangan et al. 2020), supporting the hypothesis that secondary structures are often targets of strong negative selection. The second of these studies uncovered high secondary structure conservation but low sequence conservation between SARS-CoV-2 and its close ancestor, SARS-CoV (Simmonds 2020), consistent with the observation from a comparative study between HIV-2 and SIV Berkhout (1992). Sites predicted to be base paired also demonstrated lower sequence variation than those predicted to be unpaired (Simmonds 2020), providing the first piece of evidence that disruption of base pairing is deleterious in SARS-CoV-2. Although analysis of the NMR-resolved SARS-CoV-2 secondary structure (Wacker et al. 2020) later contrasted some of these findings (Simmonds 2020), instead demonstrating both high sequence and secondary structure conservation between SARS-CoV-2 and SARS-CoV, it also showed that substitutions between these species tend to be compensatory (Wacker et al. 2020). Other studies have found high secondary structure conservation among SARS-CoV-2 and many ancestral coronaviruses (Huston et al. 2021; Sun et al. 2021), mirroring findings from early analyses not including SARS-CoV-2 (Chen and Olsthoorn 2010; Guan et al. 2012; Yang and Leibowitz 2015), as well as detected increasing sequence divergence and frequencies of compensatory substitutions with increasing phylogenetic distance (Sun et al. 2021). Intriguingly, several studies have exploited this extensive conservation of coronavirus secondary structure to predict SARS-CoV-2 functional motifs (Huston et al. 2021), interactions with host proteins (Vandelli et al. 2020; Sun et al. 2021), and putative antiviral drug targets (Aldhumani et al. 2021; Sun et al. 2021). However, whereas interest in this area is rapidly growing, research on epistasis-driven evolution of the SARS-CoV-2 RNA secondary structure currently lags far behind that on its protein tertiary structures (Castiglione et al. 2021; Rochman et al. 2021, 2022; Rodriguez-Rivas et al. 2022; Starr et al. 2022).

In this study, we take a population-level approach to assay the role of epistasis in the evolution of the SARS-CoV-2 secondary structure. Specifically, we examine several characteristics of derived alleles produced by substitution mutations at ancestral WC base-paired (WC) and unpaired (UP) sites in the NMR-resolved secondary structure of SARS-CoV-2 (Wacker et al. 2020). First we address the hypothesis that epistasis constrains evolution of the secondary structure by comparing saturation levels and derived allele frequencies between ancestral WC and UP sites. Second, we evaluate whether UP nucleotides are more deleterious than G·U wobbles at ancestral WC sites by comparing derived allele frequencies between WC→UP and WC→GU mutations. Last, we identify WC sites and topological motifs evolving under the strongest and weakest epistatic constraint by evaluating the distribution of derived allele frequencies at ancestral WC sites. Together, these analyses provide a novel framework for understanding the evolutionary trajectory of the secondary structure in an important viral pathogen.

## Results and Discussion

To investigate epistasis in the SARS-CoV-2 secondary structure, we extracted reference and alternate alleles corresponding to substitution mutations in three genomic regions containing 15 NMR-resolved conserved topological motifs (Wacker et al. 2020) (see “Methods” section for details). In particular, we considered 5116 alternate alleles at 885 positions for which the SARS-CoV-2 reference (Wuhan-Hu-1; NC_045512.2) allele is conserved in the reference genome of SARS-CoV (Tor2; NC_004718.3). Inter-species conservation of reference alleles, particularly for two rapidly evolving viruses, ensured that assayed positions have been subject to long-term constraint and are thus likely of evolutionary importance. Moreover, this conservation requirement enabled us to polarize mutations at interrogated positions by setting reference alleles as “ancestral” and alternate alleles as “derived.” Of the 5116 ascertained derived alleles, 2463 contain known (A, U, G, or C) nucleotides, yielding a mean of approximately 2.78 derived alleles per position. Given the three possible derived alleles at each position, the mean saturation across positions can be estimated as $100(2.78/3)=92.8$%. This nearly complete saturation of assayed positions is consistent with the high mutation rates of RNA viruses (Drake 1993; Lynch 2010; Duffy 2018).

If there is epistatic constraint to maintain the SARS-CoV-2 secondary structure, then we expect fewer allowable alleles at WC sites to result in lower saturation by derived alleles at WC than at UP sites. To address this question, we divided the 885 genomic positions into 352 (176 pairs) WC and 533 UP sites (Tables S1 and S2). Among the 2463 annotated mutations with known derived alleles at these positions, 931 occur at WC sites and 1532 at UP sites. Thus, on average, there are approximately 2.64 and 2.87 derived alleles per position at WC and UP sites, respectively. Again considering the three possible derived alleles at each position, WC sites are generally ~88.2% saturated, whereas UP sites are ~95.8% saturated. This difference is highly significant ($P=1.01\times 10^{-24}$, binomial test; see “Methods” section for details), consistent with the expectation of lower saturation at WC than at UP sites, and therefore with the hypothesis that there is epistatic constraint to preserve the SARS-CoV-2 secondary structure. Moreover, the similar saturation imbalance reported in HIV-1 (Assis 2014) suggests that our finding may be generalizable to other RNA viruses that have yet to be explored in this context.

A second expectation of epistatic constraint to maintain the SARS-CoV-2 secondary structure is that purging of deleterious alleles at WC sites by negative selection will result in lower derived allele frequencies at WC than at UP sites. In tackling this question, it was important to ensure complete derived allele counts by considering the full set of 5116 annotated mutations, which consisted of 1818 mutations at WC sites and 3298 mutations at UP sites (see “Methods” section for details). Further, as demonstrated by the observed saturation levels, high viral mutation rates often result in multiple derived alleles at a given position. Thus, for each position, we computed the derived allele frequency as the sum of the frequencies of all derived alleles (Tables S1 and S2; see “Methods” section for details), and then compared the distributions of derived allele frequencies between WC and UP sites (Fig. 2A). Indeed, derived allele frequencies at WC sites are significantly lower than those at UP sites ($P=2.32\times 10^{-5}$, Mann–Whitney *U* test; see “Methods” section for details). In particular, the median derived allele frequency at WC sites is ~72.2% lower than that at UP sites, suggesting that there is strong negative selection against disruption of WC base pairing. Hence, this analysis provides additional support for the hypothesis that there is epistatic constraint to preserve the SARS-CoV-2 secondary structure. Further, an analogous result in HIV-1 (Assis 2014), as well as similar conclusions reached from evolutionary studies of other RNA viruses (Berkhout 1992; Harrison and Lever 1992; Berkhout et al. 1997; Liu et al. 2009; Fernández et al. 2013), indicate that the observed population-level evolutionary trend may persist across diverse RNA viruses.

Last, epistatic constraint to maintain the SARS-CoV-2 secondary structure should result in particularly strong negative selection against alleles that abolish base pairing, and therefore lower derived allele frequencies for WC→UP than WC→GU mutations. Because the identities of alternate alleles were required to address this question, here we only considered the 2463 annotated derived alleles with known nucleotides, as we did for our saturation analyses. In particular, we computed two derived allele frequencies for each WC site: the derived allele frequency for WC→UP mutations, computed as the sum of the frequencies of all derived alleles yielding UP nucleotides, and the derived allele frequency for WC→GU mutations, computed as the sum of the frequencies of all derived alleles yielding G·U wobbles (see “Methods” section for details). Then we compared distributions between derived allele frequencies for WC→GU and WC→UP mutations (Fig. 2B). Indeed, derived allele frequencies for WC→UP mutations are significantly lower than those for WC→GU mutations ($P=1.31\times 10^{-9}$, Mann–Whitney *U* test; see “Methods” section for details). In particular, the median derived allele frequency for WC→UP mutations is ~30.7% lower than that for WC→GU mutations, indicating that negative selection against alleles generated by WC→UP mutations is much stronger, perhaps because G·U wobbles can retain much of the stability of the secondary structure. Therefore, this analysis further supports the hypothesis that there is epistatic constraint to preserve the SARS-CoV-2 secondary structure through retention of base pairing. Moreover, our findings again mirror those from an analogous study in HIV-1 (Assis 2014), and are also consistent with observations in numerous secondary structures (Rousset et al. 1991; Olsthoorn et al. 1994; Kirby 1995; Stephan 1996; Innan and Stephan 2001; Dutheil et al. 2010; Meer et al. 2010), warranting investigation of this phenomenon in populations of other RNA viruses. Finally, though not possible with the current dataset (see “Methods” section for details), it would be interesting to extend this approach to investigate the evolution of the SARS-CoV-2 secondary structure through compensatory mutations (Fig. 1) (Rousset et al. 1991; Kirby 1995; Assis 2014).

**Fig. 3:**
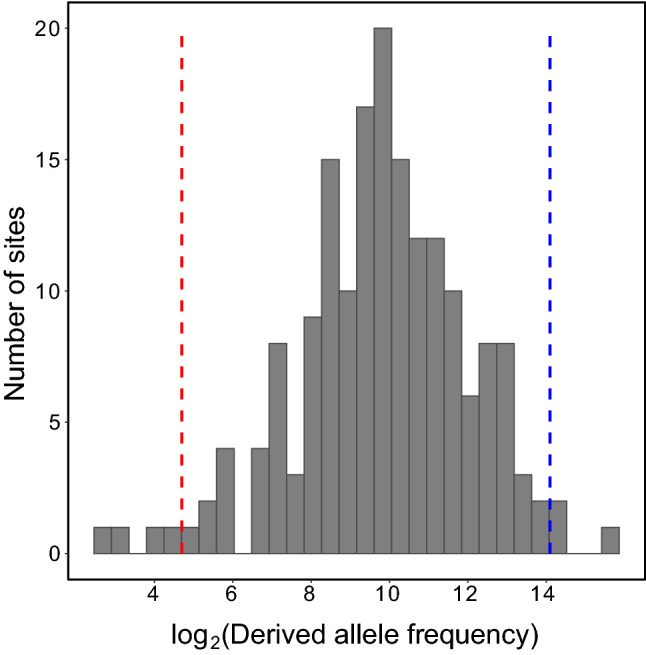
Identification of WC sites under the strongest and weakest epistatic constraint. Log-transformed derived allele frequency spectrum for ancestral WC sites. Red and blue dashed lines show cutoffs used for selecting WC sites under the strongest and weakest epistatic constraint, respectively. Table S1 provides details for these sites, which are displayed in red and blue text (Color figure online)

**Fig. 4:**
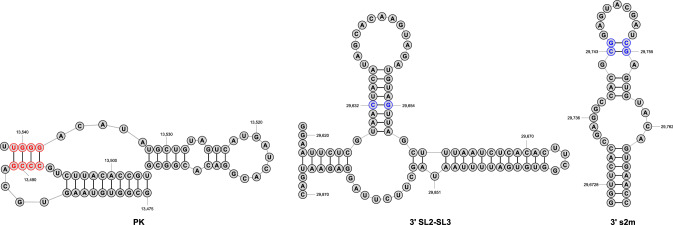
Motifs containing WC sites under the strongest and weakest epistatic constraint. WC sites colored in red and blue correspond to those from Fig. 3 that are under the strongest and weakest epistatic constraint, respectively (Color figure online)

Given the abundant support for epistatic constraint on the secondary structure of SARS-CoV-2, we next sought to identify and characterize WC sites under the strongest and weakest constraint, as such sites represent putative targets of negative and positive selection, respectively. To address this problem, we considered the distribution of derived allele frequencies at WC sites by examining the derived allele frequency spectrum (Fig. 3). We selected four sites with fewer than 20 derived alleles as those under the strongest constraint (Table S1, rows 121–124), and three sites with more than 18,000 derived alleles as those under the weakest constraint (Table S1, rows 150, 178, and 179). The four sites under the strongest constraint compose the entirety of the second stem of the pseudoknot (PK) motif (Fig. 4, left), which is centrally located in the frameshifting region of the SARS-CoV-2 genome (Wacker et al. 2020). Although these sites are also found within the ORF1ab protein-coding gene, their constraint appears to be primarily attributed to epistasis on the second stem of PK. For one, ORF1ab spans from positions 266–21,555, composing >70% of the SARS-CoV-2 genome and, consequently, containing ~48% of the WC sites examined in our study. Second, among the four sites considered here, the only site with no nonsynonymous derived alleles is located at the interior of the second stem of PK, where constraint is notably strongest. Specifically, the two interior sites have both the lowest and identical derived allele frequencies for WC→GU mutations, and both the lowest and comparable derived allele frequencies (Table S1, rows 122 and 123). Similarly, the two exterior sites have both higher and identical derived allele frequencies for WC→GU mutations, and both higher and comparable derived allele frequencies (Table S1, rows 121 and 124). This distinct palindromic pattern implies constraint to maintain the structural integrity of the second stem of PK. Indeed, the structure of this stem is highly conserved across coronaviruses (Plant et al. 2005; Su et al. 2005; Wacker et al. 2020), and mutagenesis studies have revealed it to be essential to programmed ribosomal frameshifting during ORF1ab translation (Baranov et al. 2005; Plant et al. 2005), a tightly controlled strategy that enables viral translation of multiple proteins from a single mRNA (Atkins et al. 2016). Thus, the population-level epistatic constraint observed here likely reflects the functional importance of the second stem of PK in SARS-CoV-2.

Contrary to our findings for WC sites under the strongest epistatic constraint, the three sites under the weakest constraint are all located in the 3$^{\prime }$ UTR of the SARS-CoV-2 genome. The first site (Table S1, row 150) is situated roughly in the center of the longest stem of the overlapping SL2 and SL3 motifs (Fig. 4, center). Interestingly, this site has more than double the derived allele frequency of any other site, primarily as a result of WC→GU mutations, and therefore may also be evolving rapidly via compensatory mutations. SL2 is thought to compose an RNA switch that regulates viral replication (Goebel et al. 2004; Züst et al. 2008), and SL3 is involved in long-range interactions between the 3$^{\prime }$ end of the genome and single-stranded regions flanking SL2 (Züst et al. 2008). Hence, positive selection on this WC site has the potential to simultaneously influence two structures that may play critical roles in viral replication. The other two WC sites identified (Table S1, rows 178 and 179) are located at adjacent positions that compose a small two base-pair stem in the s2m motif (Fig. 4, right) (Wacker et al. 2020). Because s2m is highly conserved across coronaviruses (Goebel et al. 2007; Wacker et al. 2020), weak epistatic constraint on these WC sites presents an intriguing puzzle. Examination of the two sites in the s2m motif (Fig. 4, right) reveals that co-occurrence of WC→UP mutations would increase the size of the neighboring loop, dramatically altering the structure and perhaps function of s2m. Although the specific function of s2m has yet to be determined, it is found within the hypervariable region (HVR) associated with viral pathogenicity (Goebel et al. 2007). Thus, positive selection on these WC sites may contribute to adaptation through optimization of SARS-CoV-2 pathogenicity. Though preliminary, these case studies suggest that negative selection may act to preserve the integrity of frameshifting, and positive selection to enable adaptive modifications of replication and pathogenicity. Further examination of these and other WC sites that may be evolving under negative and positive selection in SARS-CoV-2 can promote our understanding of how an important virus adapts to novel environments when traversing a rugged fitness landscape generated by epistatic constraint on its secondary structure.

## Methods

### Ascertainment of Polymorphism Data for Substitution Mutations

A table containing positions, identities, and intra-population frequencies of reference and alternate alleles corresponding to substitution mutations across the SARS-CoV-2 genome was downloaded from the China National Genomics Data Center (NGDC) of the China National Center for Bioinformation (CNCB) (Gong et al. 2020; Song et al. 2020; Zhao et al. 2020; Yu et al. 2022) at https://ngdc.cncb.ac.cn/ncov/variation/annotation on April 13, 2022. Data in the NGDC are derived from SARS-CoV-2 sequences deposited in several public databases, including the U.S. National Center for Biotechnology Information (NCBI) (Sayers et al. 2022). To obtain the most complete polymorphism dataset for our analysis, we set “type of mutation” = “SNP” (denoting “single-nucleotide polymorphism” mutation) and left all other fields with their default parameters, such that the search was performed across all genomic positions and available strains. At the time of retrieval (April 13, 2022), the resulting table contained 130,943 alternate alleles and their frequencies in 5,013,151 SARS-CoV-2 strains. Of these alternate alleles, 55,786 had unknown (B, D, H, K, M, N, R, S, V, W, and Y) nucleotides. To be conservative, we included these alternate alleles for analyses requiring complete counts (i.e., those presented in Figs. 2A and 3) and removed them for analyses requiring knowledge of the identities of nucleotides (i.e., comparison of site saturation and that presented in Fig. 2B).

### Identification of Ancestral and Derived Alleles at WC and UP Sites

Genomic positions of 446 (223 pairs) WC and 899 UP sites were extracted from the NMR-resolved RNA secondary structure of SARS-CoV-2 (Wacker et al. 2020), which contains 15 conserved structural motifs in the 5$^{\prime }$ end (1–471), ribosomal frameshift segment (13,434–13,541), and 3$^{\prime }$ UTR (29,548–29,867). We restricted our analyses to these regions in which the RNA secondary structure was resolved. However, because the NGDC did not contain polymorphism information for positions 1–19, we removed 14 unpaired and five paired positions from the 5′ SL1 motif. Therefore, at the corresponding paired sites (first five rows of Table S1), WC→UP and WC→GU mutations could only assayed at one position. Further, we only considered positions with at least one annotated alternate allele in the NGDC (see “Ascertainment of Polymorphism Data for Substitution Mutations” section), and at which the reference allele is conserved between SARS-CoV-2 and its ancestor, SARS-CoV (Wacker et al. 2020), such that we could assume that the reference allele has likely been under long-term constraint and represents the ancestral state for polarization. After applying these filters, our dataset consisted of 352 (176 pairs) WC and 533 UP sites. At each site, we counted the number of distinct known (A, U, G, and C) and unknown (B, D, H, K, M, N, R, S, V, W, and Y) derived alleles in the NGDC dataset. In total, there were 5116 NGDC derived alleles annotated across all sites, 1818 at WC sites, and 3298 at UP sites. Of these annotated derived alleles, 2463 had known nucleotides, 931 at WC sites, and 1532 at UP sites.

### Calculation of Derived Allele Frequencies

In our study, we chose to represent derived allele frequencies as absolute frequencies, or counts. Although derived allele frequencies are more commonly given as relative frequencies in the population, we made this decision for two reasons. First, the NGDC CNCB resource (Gong et al. 2020; Song et al. 2020; Zhao et al. 2020; Yu et al. 2022) from which the mutations in this study were obtained does not contain information on the number of SARS-CoV-2 strains with missing data at each position. Therefore, relative frequencies would be computed here by dividing all absolute frequencies by the population size, which is 5,013,151 (number of SARS-CoV-2 strains). Dividing absolute frequencies by a constant would result in proportional shifts of the distributions of values, and hence would not alter any of the observed patterns or findings. Second, counts are more interpretable than small fractions, enabling easier understanding of our findings. Thus, given that absolute and relative frequencies are interchangeable here, we decided to use absolute derived allele frequencies to enhance understanding.

Further, it is important to note that the NGDC dataset used for our study only contains polymorphisms, and not full viral sequences. Thus, it is impossible to determine which two alleles segregate together (co-occur in the same viral strains) at a particular WC site. As a result, we were unable to assay compensatory mutations (Fig. 1, bottom), and were therefore limited to the assumption that all mutations correspond to single mutations (Fig. 1, middle). Given this assumption, only one of the six possible substitution mutations at any WC site generates a G·U wobble. For example, suppose that the ancestral WC site contains a G at one position and a C at the other position (Fig. 1, top). In this scenario, the G can be replaced with a C, U, or A. Similarly, the C can be replaced with a G, U, or A. Of these six mutations, only the replacement of the C with a U creates a G·U wobble (Fig. 1, middle right). Hence, for this example, the derived allele frequency for WC→GU mutations would be computed as the number of derived U alleles at the ancestral C, and the derived allele frequency for WC→UP mutations would be computed as the total number of all other derived alleles at either position of the WC site.

### Statistical Analyses

All statistical analyses were performed in R (R Core Team 2021) with the RStudio IDE (RStudio Team 2020). A two-tailed binomial test implemented with the binom.test() function in the stats package (R Core Team 2021) was used to compare saturation between WC and UP sites. In particular, after removal of derived alleles with unknown nucleotides (see “Identification of Ancestral and Derived Alleles at WC and UP Sites” section), there were 931 derived alleles observed at 352 WC sites and 1532 at 533 UP sites. Because there are three possible derived alleles at a particular site, the total numbers of possible derived alleles across all WC and UP sites are 1056 and 1599, respectively. Thus, in our binomial test, we set the number of successes $x=931$ to represent the number of derived alleles observed at WC sites, the number of trials $n=1056$ to represent the total number of possible derived alleles at WC sites, and the probability of success $p=1532/1599$ to represent the expected probability of saturation at WC sites if it is equal to that at UP sites. Two-tailed Mann–Whitney *U* tests implemented with the wilcox.test() function in the stats package (R Core Team 2021) were used to evaluate differences between distributions of derived allele frequencies at WC and UP sites (Fig. 2A) and between derived allele frequencies for WC→UP and WC→GU mutations (Fig. 2B).

## Data Availability

The genomic variation data underlying this article are available in the China National Center for Bioinformation National Genomics Data Center SARS-CoV-2 variation annotation database at https://ngdc.cncb.ac.cn/ncov/variation/annotation. Data produced and analyzed in this study are provided in Supplementary Tables S1 and S2.

## Supplementary Information

Below is the link to the electronic supplementary material.

Supplementary file 1 (xlsx 493 KB)
